# Nano-Medicine for Thrombosis: A Precise Diagnosis and Treatment Strategy

**DOI:** 10.1007/s40820-020-00434-0

**Published:** 2020-04-20

**Authors:** Min Su, Qixuan Dai, Chuan Chen, Yun Zeng, Chengchao Chu, Gang Liu

**Affiliations:** 1 State Key Laboratory of Molecular Vaccinology and Molecular Diagnostics, Center for Molecular Imaging and Translational Medicine School of Public Health, Xiamen University, Xiamen, 361102 People’s Republic of China; 2 State Key Laboratory of Physical Chemistry of Solid Surfaces, The MOE Key Laboratory of Spectrochemical Analysis and Instrumentation, College of Chemistry and Chemical Engineering, Xiamen University, Xiamen, 361005 People’s Republic of China; 3 Department of Pharmacy, Xiamen Medical College, Xiamen, 361023 People’s Republic of China; 4 Xiamen Cardiovascular Hospital, Xiamen University, Xiamen, 361102 People’s Republic of China

**Keywords:** Thrombosis, Nano-medicine, Diagnosis, Thrombolytic therapy

## Abstract

Thrombosis is a global health issue and one of the leading factors of death. However, its diagnosis has been limited to the late stages, and its therapeutic window is too narrow to provide reasonable and effective treatment. In addition, clinical thrombolytics suffer from a short half-life, allergic reactions, inactivation, and unwanted tissue hemorrhage. Nano-medicines have gained extensive attention in diagnosis, drug delivery, and photo/sound/magnetic-theranostics due to their convertible properties. Furthermore, diagnosis and treatment of thrombosis using nano-medicines have also been widely studied. This review summarizes the recent advances in this area, which revealed six types of nanoparticle approaches: (1) in vitro diagnostic kits using “synthetic biomarkers”; (2) in vivo imaging using nano-contrast agents; (3) targeted drug delivery systems using artificial nanoparticles; (4) microenvironment responsive drug delivery systems; (5) drug delivery systems using biological nanostructures; and (6) treatments with external irradiation. The investigations of nano-medicines are believed to be of great significance, and some of the advanced drug delivery systems show potential applications in clinical theranotics.

## Highlights

- Recent advances in diagnosis and treatment of thrombosis using nano-medicine are summarized in this review.
- The diagnosis system based on biomarkers and imaging nanoprobes could enable the detection in early state of thrombosis.
- The targeted drug delivery nanosystems serve as clinically translatable theranostics for thrombosis treatment with minor side effects.

## Graphical abstract

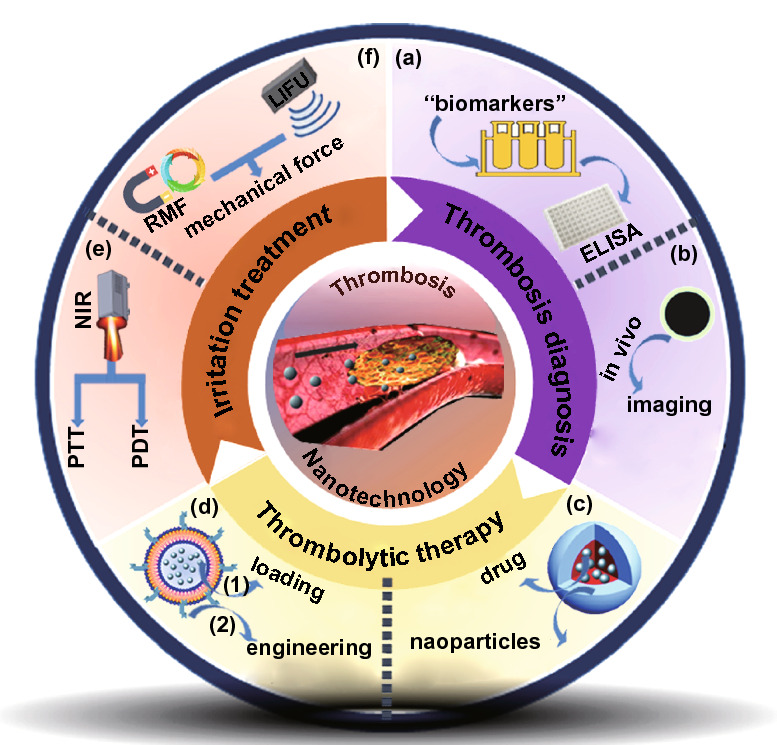

## Introduction

Thrombosis, or the formation of a malignant blood clot, is associated with many cardiovascular diseases, such as myocardial infarction and stroke. It is also one of the leading causes of death [1–5]. In thrombus formation, platelets, coagulation, and flow conditions play primary roles, and understanding these factors could provide new methods for the treatment of thrombosis [6–8]. However, the diagnosis of thrombosis is limited to late stages, and the narrow therapeutic window makes it unable to provide reasonable and effective treatment [9, 10]. Thus, the early diagnosis of thrombosis is urgently required.

The main antiplatelet and anticoagulant agents for thrombolysis treatment are heparin, urokinase plasminogen activator (uPA), tissue plasminogen activator (tPA), recombinant tPA (rtPA), and streptokinase (SK) [11, 12]. With the help of these agents, thrombus can be dissolved, or thrombus formation can be slowed down. However, these protein-based agents suffer from a short half-life, allergic reactions, or inactivation. Furthermore, due to the low accumulation efficiency and poor targeting ability, these agents have little therapeutic effect [13]. Unfortunately, this might give rise to unwanted hemorrhage in tissues or sites and induce other severe cardiovascular diseases. Thus, it is important to efficiently deliver thrombolytics to thrombus with minimum adverse effects for clinical application [14].

**Fig. 1:**
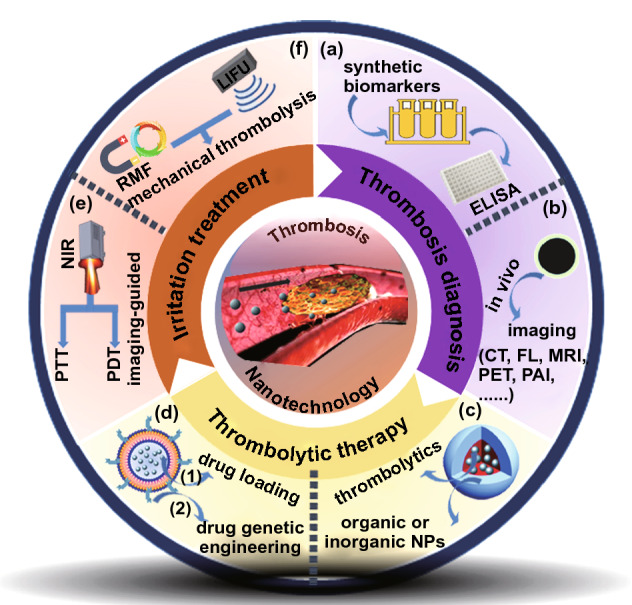
Scheme illustration of nano-medicine-based thrombosis diagnosis and treatment

In recent years, nano-medicine has gained extensive attention among scientific researchers and clinicians since the first study in the late 1990s [14, 15]. Many nanoparticle-based drug delivery systems have been investigated [16, 17], including polymeric nanoparticles (liposomes and micelles), gold/silver/platinum noble metal nanoparticles, silica nanoparticles, carbon nanostructures (graphene oxide and carbon nanotubes), up-conversion nanoparticles, and metal–organic frameworks. Many nanoparticle-based detection systems have also been constructed [14, 18]. Nanoparticles are widely studied in modern healthcare due to their convertible shape, size, physicochemical properties, and surface-area-to-volume ratio [19, 20]. Various nano-medicines have been studied in disease therapeutics, which have been proven to enhance treatment efficiency significantly and meet the requirements of individualized treatment [21–23]. Recently, Huang et al. [13] have summarized the drug delivery systems toward thrombosis treatment, with emphasize on the delivery of thrombolytics under the outside irradiation-directed targeting. In this review, we summarize the recent advances regarding to nano-medicine-based thrombosis diagnosis and treatment (Fig. 1): (1) in vitro diagnostic kits using “synthetic biomarkers”; (2) in vivo imaging; (3) targeted drug delivery systems using artificial nanoparticles; (4) microenvironment responsive drug delivery systems; (5) drug delivery systems using biological nanostructures; (6) treatments with external irradiation. In addition, we outlook for further studies to meet the demands of clinical applications.

## Nanoparticle-Based Diagnosis

### In Vitro Diagnosis

In vitro diagnosis has widely been studied for many diseases due to its advantages of simplicity, rapidness, and noninvasiveness [24, 25]. Methods using commercial 96-well plates, such as the enzyme-linked immunosorbent assay (ELISA), have been applied in hospital diagnosis. Nanoparticle-based assays in vitro have been constructed to detect biomarkers with a low detection limit for the diagnosis of diseases at an early stage [26–28]. However, the biomarkers associated with thrombosis could not be detected directly by in vitro assays because the biomarkers only exist in blood clots, and there are no prominent biomarkers in the blood.

The by-product produced when prothrombin is cleaved into thrombin is prothrombin fragment 1.2, and the by-product of fibrin degradation is D-dimer. These by-products show poor specificity in thrombosis detection [29]. Thus, the in vitro diagnosis of thrombosis cannot be achieved in an ordinary way, and the specific biomarkers for thrombosis should be investigated. The renal system could quickly and selectively filter the biological by-products from the blood, but the “synthetic biomarkers” collected from the metabolism after injection could be an effective solution [30, 31].

**Fig. 2:**
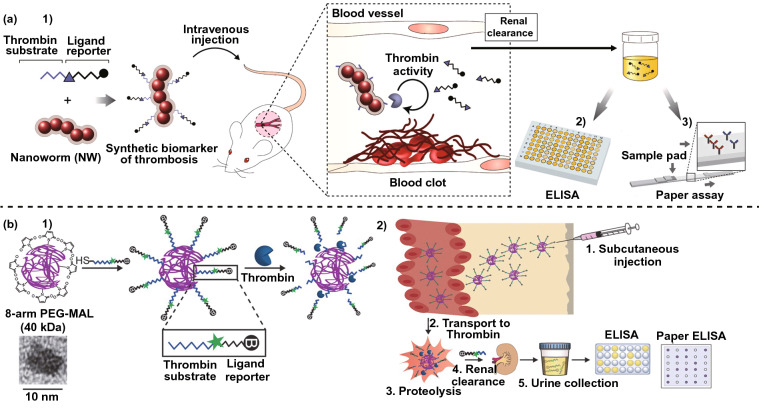
“Synthetic biomarkers” for detection of thrombus. **a** The “synthetic biomarkers” was prepared using the NW and thrombin-sensitive substrate conjugated ligand reporter (**1**); after intravenous injection of “synthetic biomarkers,” the ligand reporter was released due to the thrombin activity, the urine was then collected and detected by ELISA (**2**) or paper assay (**3**) [32]. Copyright 2013 Creative commons. **b** The “synthetic biomarkers” was prepared using 8-arm PEG-MAL and thrombin-sensitive substrate conjugated ligand reporter (**1**); (**2**) after subcutaneous injection, the urine was collected and the detection assay was carried out [34]. Copyright 2016 Wiley-VCH Verlag GmbH & Co. KGaA

Thrombin-activatable peptide (TAP) only responds to thrombin activity and is specifically cleaved by thrombin. Bhatia et al. [32] attempted to detect thrombosis using synthetic biomarkers. In the assay, iron oxide nanoworms (NWs) were prepared as a nano-carrier for the agents [32, 33]. A ligand-labeled peptide containing the TAP sequence was constructed, and peptide derivatives were modified on the surface of the NWs (Fig. 2a-1). The prepared nanocomposites showed selectivity to thrombin, and the peptide fragment was released from the nanocomposites under thrombin activity.

**Fig. 3:**
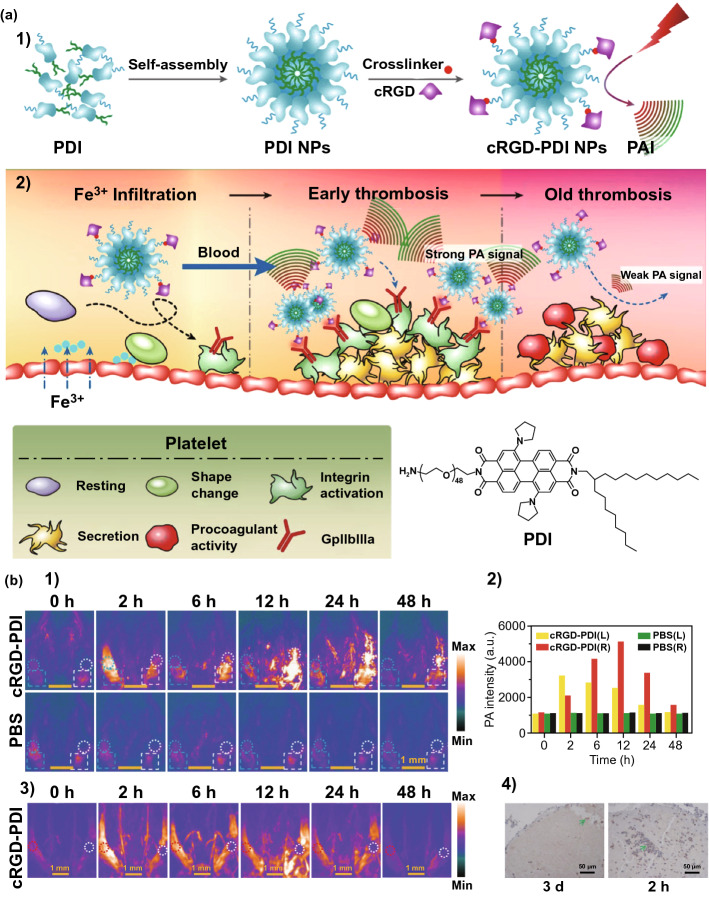
**a** The preparation of the cRGD–PDI nanoparticles (**1**); the cRGD–PDI nanoparticles could accumulate in the early thrombus for diagnosis by PAI (**2**). **b** PAI (**1**) and PA (**2**) intensity of the thrombus mouse model after different treatments (normal vessel, left vein; vessel with thrombus, right vein); (**3**) PAI of normal vessel (left vein) and old venous thrombus (right vein) after the administration of cRGD–PDI nanoparticles; (**4**) GPIIb/IIIa expression of old thrombus and early thrombus [53]. Copyright 2017 American Chemical Society

After the nanocomposite and thromboplastin were co-injected into mice intravenously, urine was collected, and the dispersed peptide fragment was detected in vitro using ELISA (Fig. 2a-2) or a paper assay (Fig. 2a-3). This kind of thrombin-cleavable fluorogenic probe could also be applied to fluorescent imaging of the localized blood clots in vivo, and the intensity attenuation reveals the thrombosis. However, the assay requires metabolism in vivo for a long time and could not realize the rapid determination for thrombosis. Furthermore, the administration route of intravenous injection also limits the possibility of home-supplied point of care.

To realize the synthetic biomarker test strategy with fast speed to enable facile monitoring of at-risk patients, Bhatia et al. [34] conjugated the peptide derivative to the surface of a poly(ethylene glycol) scaffold (PEG-T1E) to prepare another synthetic biomarker nanocomposite (Fig. 2b-1). The hydrodynamic diameter of PEG-T1E (40 kDa) was ~ 8 nm, and it could promote the subcutaneous delivery of the nanocomposite into the bloodstream. In addition, the urine signal after subcutaneous administration of the synthetic biomarkers was increased for 3 h and maintained for hours.

When the biomarker was subcutaneously injected into mice, and thromboplastin was administered 2 h later, the in vitro assay could clearly distinguish the thromboplastin-treated mice (Fig. 2b-2). The test strategy could help to monitor for postoperative complications, in which thrombosis might delay the diagnosis. With further development, this synthetic-biomarker urine diagnosis strategy might be applied clinically and support the accurate diagnosis and location of the thrombus.

### In Vivo Imaging

Many imaging modalities have been applied in thrombus detection, including ultrasound (US) imaging [35], magnetic resonance imaging (MRI) [36], and positron emission tomography (PET) [37]. However, conventional imaging only shows applicability to aged clots, and the early diagnosis and evaluation of thrombosis are still in great demand. The visualization of biomarkers associated with thrombosis is fundamental to modern molecular imaging, and further investigation is necessary for precise treatment. There are many biological features, such as fibrin, activated platelets, and factor XIII, which are closely related to the formation of thrombosis. The associated biomarkers can act as targeting molecules for diagnosis [36]. With the development of thrombosis diagnostic techniques, various contrast agents have been produced, and the MR probe EP-2104R (fibrin-targeted agents) has already entered clinical trials [38, 39].

Furthermore, nanoparticles could diffuse into thrombi selectively. Combined with the excellent imaging performance of nanoparticles, nanoparticle-based contrast agents containing a targeting molecule might provide accurate detection of thrombosis. For example, magnetic nanoparticles have been widely studied on account of their outstanding MRI contrast properties [40–42], and Food and Drug Administration (FDA) has approved Feridex@ (a kind of magnetic nanoparticle) for the use in MRI diagnosis. The magnetic nanoparticle-based MRI diagnosis of thrombus has been investigated:

1. Previous studies have confirmed that several sulfated polysaccharides such as fucoidan could bind to P-selectin, which is involved with the intraluminal thrombus [43]. Ultrasmall superparamagnetic iron oxide nanoparticles (USPIOs) were modified with fucoidan and maintained high T2 relaxation [44]. The contrast agent was applied to detect platelet-rich thrombi, and its selectivity to the thrombus area with rapid speed was confirmed.
2. The cyclic Arg-Gly-Asp (cRGD) peptide was reported to target the activated platelets due to the surface-overexpressed GPIIb/IIIa receptor [45]. The cRGD peptide was decorated on the surface with Fe_3_O_4_-poly(lactic-*co*-glycolic acid) nanoparticles (Fe_3_O_4_@PLGA) [46]. The coated PLGA layer provided the nanoparticles with excellent biocompatibility for cardiovascular diseases. After injection with nanoparticles, the T2 signal of the area decreased after 10 min and then slightly increased until 50 min. The result demonstrated that the surface cRGD peptide enabled the accumulation of MRI contrast agents in the mural thrombus, regardless of the high shear stress.
3. Single-chain antibody (scFv) was also applied to target the activated GPIIb/IIIa receptors. After scFv was conjugated on the surface of T1/T2 dual-contrast magnetic nanoparticles, dual MR contrast was applied to thrombus imaging. After the targeted nanoparticles were injected into model mice, the T1 signal was enhanced, and the T2 signal was decreased and varied over time. The use of dual MR images by one contrast agent could help enhance the accuracy of the diagnosis.

CT imaging has difficulty in distinguishing a thrombus from adjacent blood. High-Z elements can absorb X-rays, so many materials have been designed as CT contrast agents, including gold (Au), bismuth (Bi), and zirconium (Zr)-based nanostructures and iodine (I)-containing nanostructures [47–49]. In addition, glycol chitosan (GC) Au nanoparticles (GC-AuNPs) were confirmed to show a tendency to accumulate in thrombus area [50]. For further investigation, fibrin-specific peptide (EP-2104R) was conjugated to the surface of GC-AuNPs [51]. The recurrent thrombosis could be diagnosed in 3 weeks with the fib-GC-AuNP-based microCT (mCT) imaging. Notably, the mCT imaging could be obtained after the fib-GC-AuNPs were intravenously injected for 5 min, implying fast imaging speed of this strategy. This result is of great significance for clinically personalized thrombolytic therapy.

**Fig. 4:**
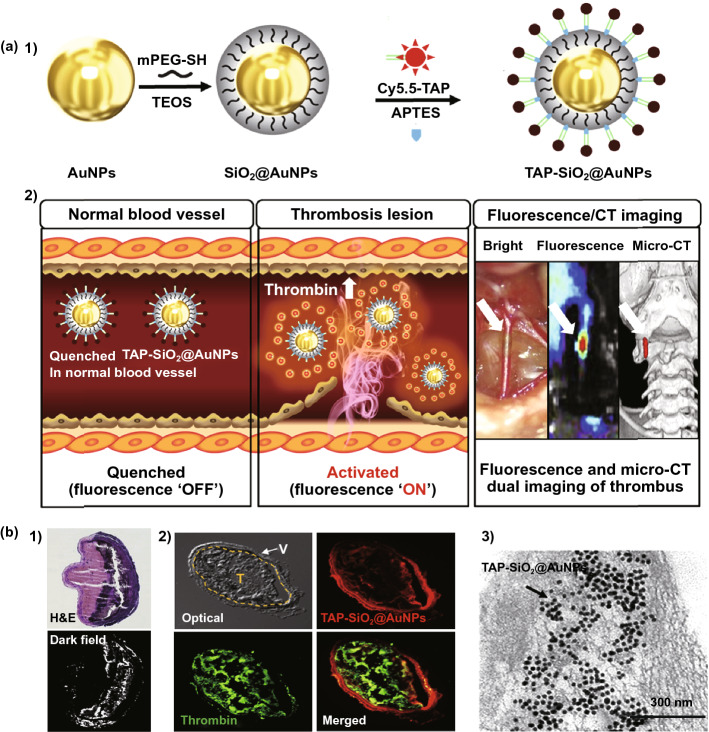
**a** Preparation procedure of TAP-SiO_2_@AuNPs (**1**); the fluorescence of the multifunctional nanoparticles was quenched, the fluorescence was activated in the thrombosis lesion, the fluorescence/CT dual imaging strategy could be applied for the diagnosis of thrombosis (**2**). **b** The H&E staining/dark field images (**1**), immunofluorescence images (**2**) and tissue TEM (**3**) of dissected-CCA from the TAP-SiO_2_@AuNPs-treated thrombotic model [58]. Copyright 2018 Elsevier Ltd

Photoacoustic imaging (PAI), a non-ionizing imaging modality, has been applied in disease diagnosis [52]. In 2017, Cui et al. [53] prepared amphiphilic perylene-3,4,9,10-tetracarboxylic diimide (PDI) derivatives and assembled them into organic semiconducting nanoparticles (Fig. 3a). The diameter of the nanoparticles was about ~ 40 nm, and the hydrate particle size was about ~ 70 nm, which is suitable for thrombosis imaging. The nanoparticles showed PA contrast properties when irradiated at 700 nm. PAI was studied after injection with the cRGD–PDI nanoparticles. As shown in Fig. 3b-1, 2, the cRGD–PDI nanoparticles could distinguish an early thrombus from healthy vessels, demonstrating good imaging properties. Furthermore, the cRGD–PDI nanoparticles could distinguish an early thrombus from an old thrombus (Fig. 3b-3). The capacity to display detailed information about an early thrombus could help in understanding of the thrombolytic process. The results confirmed the difference in GPIIb/IIIa expression between the early and old thrombus (Fig. 3b-4).

However, most of the nano-agents accumulate in liver and other organs, and the strategies might be limited to a specific type of thrombosis. Many efforts have been made to microenvironment-responsive contrast agents for cancer detection [54–56], and the effects are also appropriate for thrombosis diagnosis. Thrombin-activatable peptide, which can only be cleaved by thrombin, is a good choice for a thrombosis-responsive imaging system. In 2017, Lux et al. [57] constructed a kind of thrombin-sensitive activatable cell-penetrating peptide (ACPP) containing thrombin-sensitive peptide fragment and cell-penetrating peptide sequence. The peptide was further conjugated to US molecular perfluorobutane-filled microbubbles (MBs). In blood clots, when the ACPP-MBs are cleaved by thrombin, the ACPP is exposed on the surface of the MBs. Thus, the MBs adhere to the thrombus. When treated with thrombin-rich blood clots, the ACPP-MBs showed enhanced US imaging, demonstrating potential for acute diagnosis.

In 2018, Kwon et al. [58] constructed a fluorescent-switch system using TAP and silica-coated AuNPs (SiO_2_@AuNPs). As shown in Fig. 4a-1, SiO_2_@AuNPs were prepared, and the surface was modified with TAP. The fluorescence of TAP (Cy5.5 in the system) was quenched due to the distance-dependent quenching effect of SiO_2_@AuNPs. In the presence of thrombin, the TAP was cleaved, and the fluorescence could recover. Combined with micro-CT imaging of AuNPs as the inner nanoparticle, the TAP-SiO_2_@AuNPs could be used to detect thrombosis using dual-fluorescence and micro-CT imaging (Fig. 4a-2). Furthermore, the fluorescence imaging and a micro-CT imaging could be co-localized using the TAP-SiO_2_@AuNPs. Treated common carotid artery (CCA) tissue was dissected from a thrombotic model. As shown in Fig. 4b, dark field images showed bright signals of the gold particles. The tissue showed a fluorescence signal of the released Cy5.5. The TEM image confirmed that the nanoparticles were successfully accumulated within the thrombus tissue. In the dual-mode thrombus imaging system, the thrombus accumulation depended on the appropriate particle size of the TAP-SiO_2_@AuNPs.

The diagnosis of thrombosis using nano-medicines could be summed up in two ways. (a) In vitro diagnosis should rely on synthetic biomarkers, which can be collected in urine after the thrombosis-responsive enzyme digestion reaction and the following metabolism. This strategy provides feasibility for home-supplied point of care. The assay requires three important factors: (1) The nano-carriers should accumulate or be selectively enriched in blood clots, and the nano-medicine should have low hematotoxicity, low organ accumulation, and long half-life. (2) The detection biomarkers should be metabolized from kidney, and during these procedures the molecule should maintain its structure. (3) The detection biomarkers were modified on the nano-carriers through the connection of TAP or other thrombosis-response peptides and could be cleaved quickly. Even though the in vitro diagnosis mis-displays the detailed information about thrombosis, the simultaneous synthetic biomarkers assay has great potential for clinical application. (b) Diagnosis with nanostructure-based imaging in vivo has been the most studied approach in preclinical and clinical applications. Imaging in vivo requires two important factors: (1) the nanoparticle-based contrast agents should have outstanding imaging properties or show imaging in the microenvironment of thrombosis. (2) Surface modification should enable contrast agents long circulation time, thrombus targeting, and appropriate biosecurity. Both diagnosis strategies need further investigations for the precise detection of the various kinds of thrombosis and guiding therapy.

## Nanostructure-Based Drug Delivery

### Artificial Nanoparticles-Based Drug Delivery

Nano-medicine-based treatment for thrombolysis has also been considered. It has been applied in thrombosis and has gained higher therapeutic efficiency than pure drugs. Various drug delivery systems have been constructed to deliver drugs to thrombotic sites with reduced adverse effects, including specific targeting strategies, as well as US, magnetic, or light-driven strategies [13]. Meanwhile, the easy encapsulation of thrombolytic agents and surface targeting modification have made liposomes preferred candidates for thrombolysis [59–62]. In addition, the combined utilization of external irradiation could show effective release in thrombotic sites, as in the case of echogenic liposomes (ELIP) [63]. ELIP was prepared using a lipid monolayer shell, encapsulated with octofluoropropane and loaded with rt-PA to construct an acoustically activated drug-delivery system [64]. When the nanocomposite was injected, the surface-modified targeting molecule led to enrichment in blood clots, and then local external US application enabled the release of rt-PA and incited cavitation activity.

There are many other polymer-based thrombolytic agent-delivery systems [43, 65, 66]. Colasuonno et al. prepared a kind of erythrocyte-inspired discoidal nano-construct using PLGA and PEG, and the unique structure could prevent drug accumulation within the brain and reduce cerebral hemorrhages. tPA molecules were conjugated on the surface of the material through interaction with the PLGA chains. The unique erythrocyte-mimicking structure provided the nanocomposites long-term circulation in vivo and good blood-clot-dissolving efficiency [67].

Aside from thrombolytic agents, gene therapeutic agents have also been applied for thrombolysis. For this propose, a recombination hirudin plasmid (pDNA) was constructed with the RGD and HV genes and linked by the coagulation factor Xa (FXa) gene [68]. Furthermore, RGDyC and PEG were modified on polyamide dendrimer (PAMAM). With the help of surface-modified RGDyC, pDNA could target thrombotic sites, and recombinant hirudin fusion protein was expressed. The RGDyC of the protein then induced a second targeting effect for thrombosis treatment. Magnetic nanoparticles could also show thrombolysis when loaded with thrombolytic agents [69]. Simultaneously, magnetic nanoparticles can respond to an external magnetic field and enable magnetically targeted drug delivery [70, 71].

With further researches, more organic or inorganic nanostructures could be applied in thrombosis. The design of these direct transport systems should consider three factors: (1) the morphology, size, and surface modification of the nanostructure should help the drug accumulate in blood clots, and the nanostructure itself should have low biotoxicity. (2) The loading capacity of the thrombolytic agents should match the requirements of thrombosis with minor nano-carriers, and the thrombolytic agents should not be released from the nano-carriers before the accumulation. (3) The loading or modification of contrast agents on the nano-carriers in the multifunctional nanoparticles could help with the understanding of the therapy procedure.

### Microenvironment Responsive Drug Delivery

Accurate drug release based on the disease microenvironment (DMV) has widely been studied. A DMV-responsive drug delivery system could reduce the drug release in healthy tissues [72–76]. The strategy for preferred release in the area of the lesion is very suitable for the delivery of thrombolytic agents to reduce side effects. During the formation of a thrombus, hydrogen peroxide (H_2_O_2_) plays an essential role in the platelet activation and stimulates additional platelet recruitment [77, 78]. Therefore, H_2_O_2_ is an important biomarker of activated platelets, and an H_2_O_2_ probe could separate the activated platelets from the normal platelets. The depletion of H_2_O_2_ could help prevent the activation and aggregation of platelets, thus showing antithrombotic therapeutic efficiency.

**Fig. 5:**
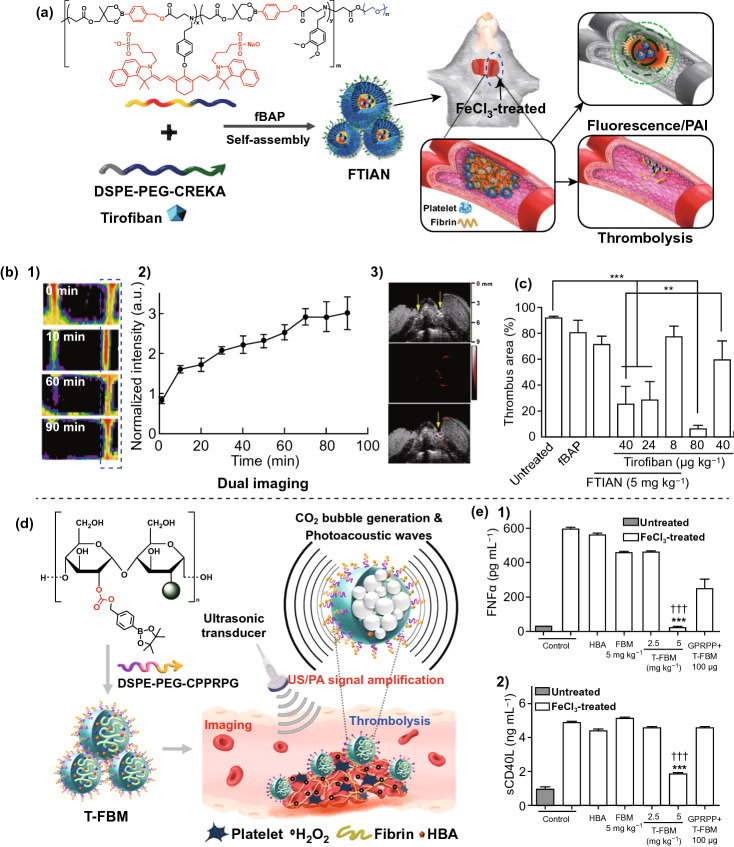
**a** Preparation diagram of FTIAN, and its application in H_2_O_2_-responsive thrombolysis. **b** Fluorescence imaging (**1**) and fluorescence intensity (**2**) of the thrombotic model after the FTIAN administration; the US/PAI of the thrombotic model after the FTIAN administration (**3**). **c** The relative thrombus size after different treatments [79]. Copyright 2017 American Chemical Society. **d** Preparation diagram of T-FBM nanoparticle, and its application in H_2_O_2_-triggered thrombolysis. **e** The expression of TNF-α (**1**) and sCD40L (**2**) of the thrombotic model after the T-FBM administration [80]. Copyright 2018 American Chemical Society

For the application of H_2_O_2_-responsive thrombolysis, Lee et al. [79] prepared a fibrin-targeted imaging and antithrombotic nano-medicine (FTIAN). FTIAN contains fibrin-targeting lipopeptides on its surface, H_2_O_2_-responsive borate, and fluorescent IR820 covalent bind polymer, as well as thrombolytic agents (tirofiban) as the inner nano-medicine (Fig. 5a). FTIAN could depolymerize after the addition of H_2_O_2_, and the tirofiban was then released. At the same time, the reduction in H_2_O_2_ could downregulate the tumor necrosis factor-alpha (TNF-α) and soluble CD40 ligand (sCD40L) of the activated platelets. Thus, FTIAN showed antioxidant and anti-inflammatory effects that were beneficial for anti-thrombogenesis and thrombolysis. As shown in Fig. 5b, the injured artery of a thrombotic model showed stronger fluorescence and PA signals than the untreated control group, and dual imaging demonstrated the thrombus-specific targeting of nanoparticles. FTIAN was confirmed to suppress the clot formation, and the thrombolysis efficiency was far higher than that of the pure tirofiban (Fig. 5c). Furthermore, FTIAN without tirofiban also showed slight thrombolysis, implying that the reduction in H_2_O_2_ could be beneficial for thrombolysis. In another study, the same group prepared fluorescent-dye (IR780) conjugated boronated maltodextrin (FBM). Thrombus-specific T-FBM nanoparticles were constructed by a self-assembly procedure [80]. As shown in Fig. 5d, when T-FBM was treated with H_2_O_2_, hydroxybenzyl alcohol (HBA) and CO_2_ were produced after several reactions. The HBA had anti-oxidant, anti-inflammatory, and anti-platelet activities, thus suppressing the thrombus formation. The produced CO_2_ showed US signals, and the PA signals of IR780 were enhanced. After the administration of T-FBM nanoparticles, the FeCl_3_-treated carotid arterial injury could clearly be distinguished by fluorescence imaging and PA imaging. Simultaneously, the TNF-α and sCD40L of the injured artery were suppressed during this procedure, confirming the antithrombotic efficiency of T-FBM nanoparticles (Fig. 5e).

In some thrombus tissues, such as ischemic brain tissue, microenvironment becomes weakly acidic due to anaerobic glycolysis [81]. pH-triggered drug release is also a strategy for selective thrombolysis treatment without influencing normal tissues. In 2019, Li et al. prepared uPA conjugated oxidized dextran (Oxd) through the connection of a pH-sensitive imine, and the RGD peptide was further connected with Oxd to provide the conjugates thrombus-targeting capacity. The uPA-Oxd could resist enzymatic hydrolysis in vivo and displayed higher activity than free uPA. Under the weakly acidic conditions of thrombus, the imine bond was hydrolyzed, and the uPA was released. The released uPA had enzymatic activity after these procedures, resulting in pH-triggered local thrombolytic therapy. Thrombin release is a critical event in thrombosis, and its high selectivity means that it could be used as a specific trigger in a thrombosis-responsive delivery system. The system could be realized with the reasonable design of TAP-containing or thrombin-responsive molecules [82]. For this purpose, Li et al. [83] packaged tPA through the in situ polymerization of acrylamide (AAm), N-(3-aminopropyl) methacrylamide hydrochloride (APM), and TAP with acrylate end groups. The polymerization helped the tPA nano-medicine hide its physiological activator property in the blood environment. The tPA nano-medicine could decompose in the presence of thrombin, and the released tPA had fibrinolytic activity, thereby dissolving blood clots.

Compared with a direct delivery system, a thrombosis-responsive delivery system could significantly reduce the side effects of thrombolytic agents. A responsive delivery system should also contain important factors that provide nano-medicines high accumulation in the thrombus tissue. Furthermore, the responsive design should strongly associate with the thrombosis microenvironment and specifically release the agents in the thrombus tissue without damages to normal tissues. A responsive drug release strategy might simultaneously change the microenvironment, resulting in a combined therapeutic effect. Even though a thrombosis-responsive delivery system is more complicated than a direct delivery system, the enhanced biosecurity provides more competitiveness for clinical applications.

### Biological Nanostructure-Based Drug Delivery

Other than organic or inorganic nanostructures, a number of biomimetic nanoparticles have been applied in drug delivery systems for disease treatment [84, 85]. Among them, cell membrane nanoparticles obtained from natural cells have been widely studied due to their high biocompatibility, long circulation, and genetic engineering modification [86–90]. Platelets play a vital role in the formation of thrombosis [91, 92], and platelet membrane (PM)-based nanoparticles have been prepared and applied for thrombosis treatment [93].

**Fig. 6:**
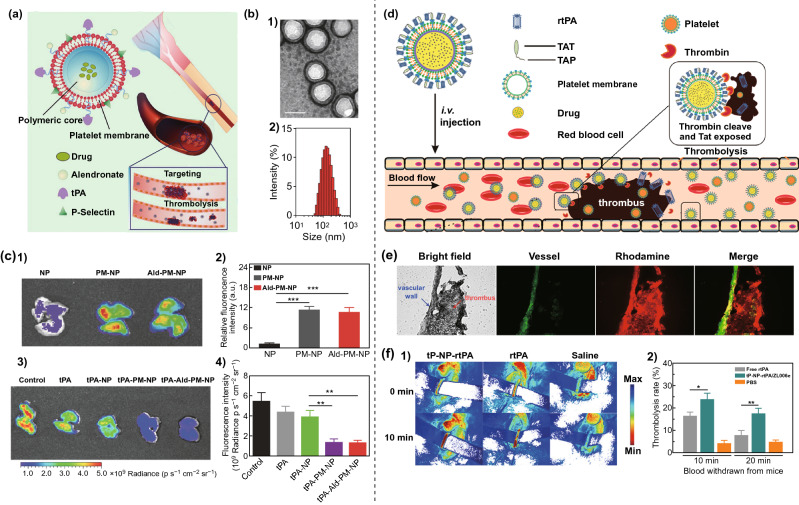
**a** Schematic illustration of tPA-PM-NP, and the surface PM enabled the targeting thrombolysis. **b** TEM (**1**) and dynamic light scattering (**2**) of tPA-PM-NP. **c** Cy5.5 labeled nanoparticles were treated with thrombus model, and the in vivo fluorescence imaging (**1**) and the fluorescence intensity (**2**) of lungs were investigated; the thrombus fluorescence imaging (**3**) and the intensity (**4**) of different treatments [94]. Copyright 2016 Wiley-VCH Verlag GmbH & Co. KGaA. **d** Schematic illustration of tP–NP–rtPA, and its thrombolysis effect. **e** Immunofluorescence images of rhodamine-labeled tP–NP–rtPA to FeCl_3_-induced carotid artery clot. **f** The bloodstream of the FeCl_3_-induced carotid artery after different treatments (**1**); the thrombolysis rate of different treatments (**2**) [95]. Copyright 2019 American Chemical Society

In 2016, Hu et al. [94] decorated the surface of PM-based nanoparticles with tPA to prepare nano-thrombolytics. As shown in Fig. 6a, the tPA was modified on the surface of the PM by a coupling reaction, and the inner polymeric core could be loaded with drugs. The PM could coat the surface of the polymeric core, and the prepared tPA–PM–NP was about ~ 127 nm (Fig. 6b). When used to treat a lung thrombosis model, the PM-modified nanoparticles showed excellent capability to target the lungs (Fig. 6c), which could be beneficial in thrombolytic therapy. Furthermore, the surface tPA could maintain the enzymatic activity after circulation for a long time. tPA–PM–NP and drug-loaded nanoparticles were injected into a lung thrombus model, and the fluorescence intensity of the fibrinogen was obviously reduced (Fig. 6c). These results confirmed that PM-based nanoparticles could home in on the thrombus site and showed a remarkable thrombolysis effect. In another study, Xu et al. [95] modified rtPA on the surface of PM through connecting with a TAP-linked cell-penetrating peptide (TAT). As shown in Fig. 6d, a drug could simultaneously be encapsulated in the multi-functional nanoparticles (tP–NP–rtPA) for a combination therapy to treat ischemic stroke. The PM and rtPA of the tP–NP–rtPA could accumulate in the thrombus site (Fig. 6e), and the bloodstream of the damaged carotid artery could recover (Fig. 6f). These results confirmed the targeted thrombolysis capability of multi-functional nanoparticles.

Furthermore, when the nanoparticles were used to treat a thrombus, rtPA was released from the nanoparticles synchronously with the exposure of TAT, and further drug delivery was realized. Yang et al. [96] loaded uPA and gold nanorods (AuNRs) in a PM nanoparticle. Unlike previous surface modification strategies, the uPA was loaded in the platelet membrane in this system, which could reduce the potential for hemorrhage. When the nanoparticles targeted the thrombosis, the inner uPA could be released from the nanoparticles in a sustained manner, resulting in an obvious thrombus decrease. Proteins and viruses have also been applied in biomimetic-based delivery systems [97, 98]. In 2017, Pitek et al. [99] loaded streptokinase (STK, a kind of thrombolytic) on the elongated tobacco mosaic virus (TMV). Due to its unique elongated geometry, the TMV-based platform had a high margination rate towards the vessel walls. As a result, the STK-modified TMV could gather in thrombotic sites under blood flow, and the STK showed effective activity.

Aside from chemical surface modification strategy, genetic engineering methods have also been an important modification strategy for delivery systems. One example is ferritin, which is well-known as a protein nanocage made of 24 subunits. Each subunit could be engineered for modification on the C-terminal or N-terminal [100]. Ferritin can be applied for drug loading by disassembly and reassembly procedures through changes of pH [101–103]. In 2018, Seo et al. [104] engineered a subunit of short ferritin with blood-clot-targeting peptides (CLT) on its N-terminal and the thrombolytic microplasmin (μPg) on its C-terminal. The CLT on the ferritin could target fibrin–fibronectin complexes, providing a thrombus-targeting property to the engineered ferritin. The μPg can be pre-activated by urokinase on active microplasmin (μPn). The ferritin can protect the μPn from the enzymatic action of α_2_-antiplasmin. Given the advantages of double functional engineering, the constructed ferritin can selectively target blood clots and shows thrombolytic/clot-busting activity.

Compared with organic or inorganic nanostructure-based delivery systems, the biomimetic strategy might show more biocompatibility for in vivo thrombolysis. Genetically engineered nanocages are uniform in size, and their thrombolytic-carrying efficiency is controllable. However, the biomimetic nanoparticles also need further investigations to meet the requirements of clinical application.

## Treatments with External Irradiation

Aside from treatments with thrombolytics, treatments based on external irradiation with the response of mechanical stress, hyperthermia, and ROS have also been studied [105]. With the help of imaging in vivo, irradiation in only thrombotic sites could reduce damages to normal tissues. For example, US imaging has been extensively applied in the clinical diagnosis of thrombosis due to its safety, real-time image monitoring, deeper penetration, and other advantages [106]. Microbubble (MB)-enhanced US can show sono-thrombolysis efficacy. Combined with the loading of thrombolytics, an US-induced fixed-point drug release system can be achieved [107, 108]. In addition, low-intensity focused US (LIFU) only reversibly modulates region-specific tissues and can meet the requirements of the irradiation area for thrombosis treatment [109]. With the use of perfluorohexane (PFH)-containing nanoparticles, LIFU could induce a liquid-to-gas phase transition (PT) procedure, and the volume expansion and explosion of the nanoparticles could damage the surrounding cells or tissues.

**Fig. 7:**
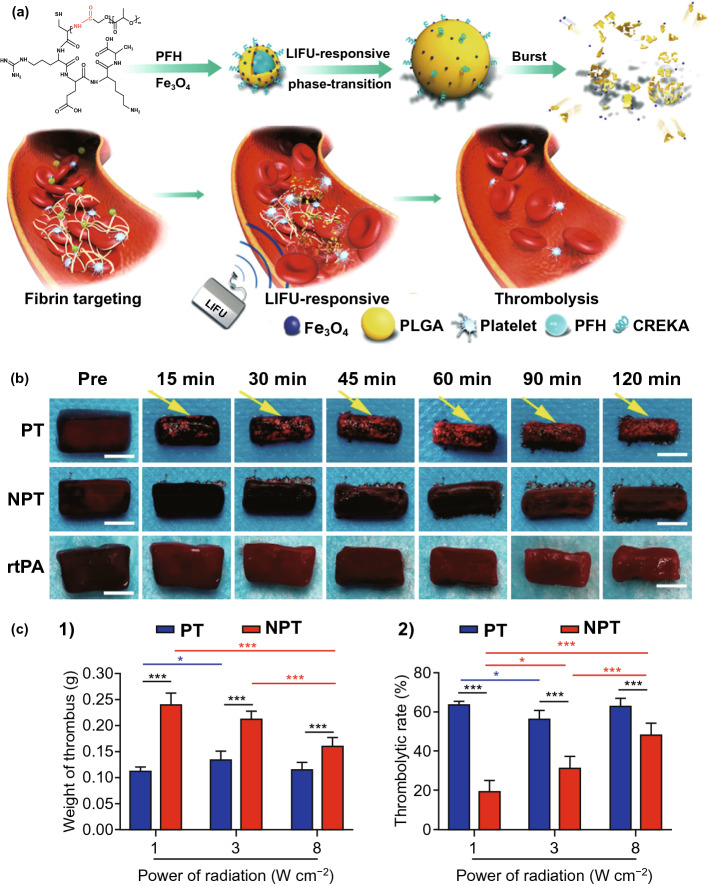
**a** Schematic illustration of PLGA-based multifunctional nanoparticles, and the PT thrombolysis process under the LIFU pulses. **b** The photographs of blood clots treated with PT, NPT, and rtPA at different time points. **c** The final thrombus weights (**1**) and final thrombolytic rates (**2**) of PT and NPT under different LIFU pulses power [110]. Copyright 2019 American Chemical Society

In 2019, Zhong et al. [110] enclosed PFH droplets in PLGA nanoparticles, and Fe_3_O_4_ nanoparticles were loaded on the surfaces of the nanoparticles (Fig. 7a). With further modification of targeting CREKA peptide, a multi-functional PT thrombolysis nano-medicine was constructed. When the nano-medicine was administered, the nanoparticles could accumulate at thrombosis, and then the LIFU irradiation was applied. As a result, the blood clots underwent hemolysis of the blood cells, fibrin degradation, and self-deformation. Nanoparticles without PFH, as a control, were also treated with LIFU irradiation (NPT). The LIFU-responsive PT thrombolysis strategy could reduce the size of the blood clots (Fig. 7b, c). The multi-functional nanoparticles also had MR, PA, and US contrast properties, thus enabling multimodal imaging in vivo to diagnose the thrombosis and monitor the PT thrombolysis efficiency. Furthermore, this nonpharmaceutical strategy had lower hepatotoxicity and risk of bleeding.

**Fig. 8:**
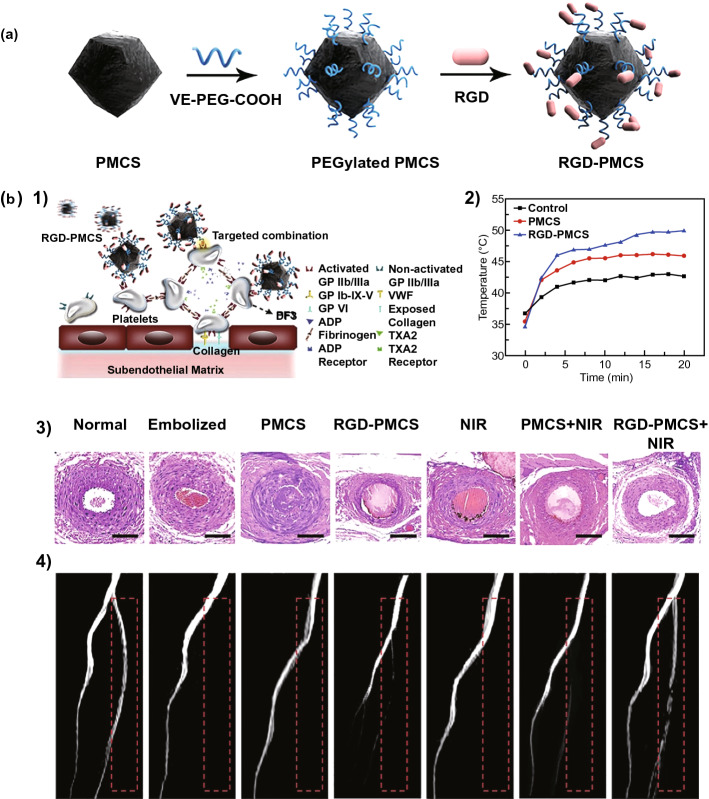
**a** Schematic illustration of RGD-PMCS. **b** Schematic illustration for thrombus specific targeting of RGD-PMCS (**1**); the temperature of treated limb of mouse after the administration of PBS, PMCS, and RGD-PMCS and irradiated by 808 nm NIR laser (**2**); H&E staining images (**3**) and MRI (**4**) of the treated limb after different treatments [117]. Copyright 2019 Creative commons

Photo-therapy (photothermal therapy, PTT; photodynamic therapy, PDT) has also been developed as a noninvasive strategy for the treatment of multiple diseases [111–114]. Photo-therapy agents can convert laser radiation into a local temperature increase or reactive oxygen species (ROS), leading to hyperthermic injury of the treated cells [115]. ROS or hyperthermia can also destroy red cells, platelets, or fibrin, meaning that photo-therapy could be applied for blood clot lysis [116]. In 2019, Zhang et al. [117] prepared mesoporous carbon nanospheres (PMCSs) from a metal–organic-framework precursor, which showed both PTT and PDT properties due to their porphyrin-like metal centers. Then, RGD was modified on the surface of the MCS to target a thrombus (Fig. 8a). When RGD-PMCS was intravenously injected into a mouse model of thrombus, it accumulated in the thrombosis, and the temperature of the thrombotic sites was noticeably increased under local irradiation with an 808-nm laser (Fig. 8b). After the PTT/PDT combined treatment, platelet factor 3 (PF3) was damaged, and red cells and the fibrin skeleton of the blood clots underwent apoptosis and broken, respectively. Thus, the PTT/PDT combined thrombolytic therapy showed an outstanding therapeutic effect, and secondary embolism was prevented thanks to the full breakage of the blood clots.

An external laser could be applied to control drug delivery. For an NIR-responsive thrombolytic release system, Wang et al. [118] prepared gold@mesoporous silica core–shell nanospheres (Au@MSNs), and uPA was co-delivered with 1-tetradecanol (Tet) into the Au@MSNs. The liquid form of Tet was transformed to a solid form when the temperature decreased. Thus, the uPA was capped into the pores of Au@MSNs, providing steady loading. The constructed loading system had stability in the cell culture medium, even after 7 days. When the nanocomposite was gathered in the thrombotic site, NIR laser irradiation was performed. The inner gold nanoparticles showed a PTT effect, and once it rose to 39 °C the Tet was reverted to a liquid form, inducing the fast release of uPA.

**Fig. 9:**
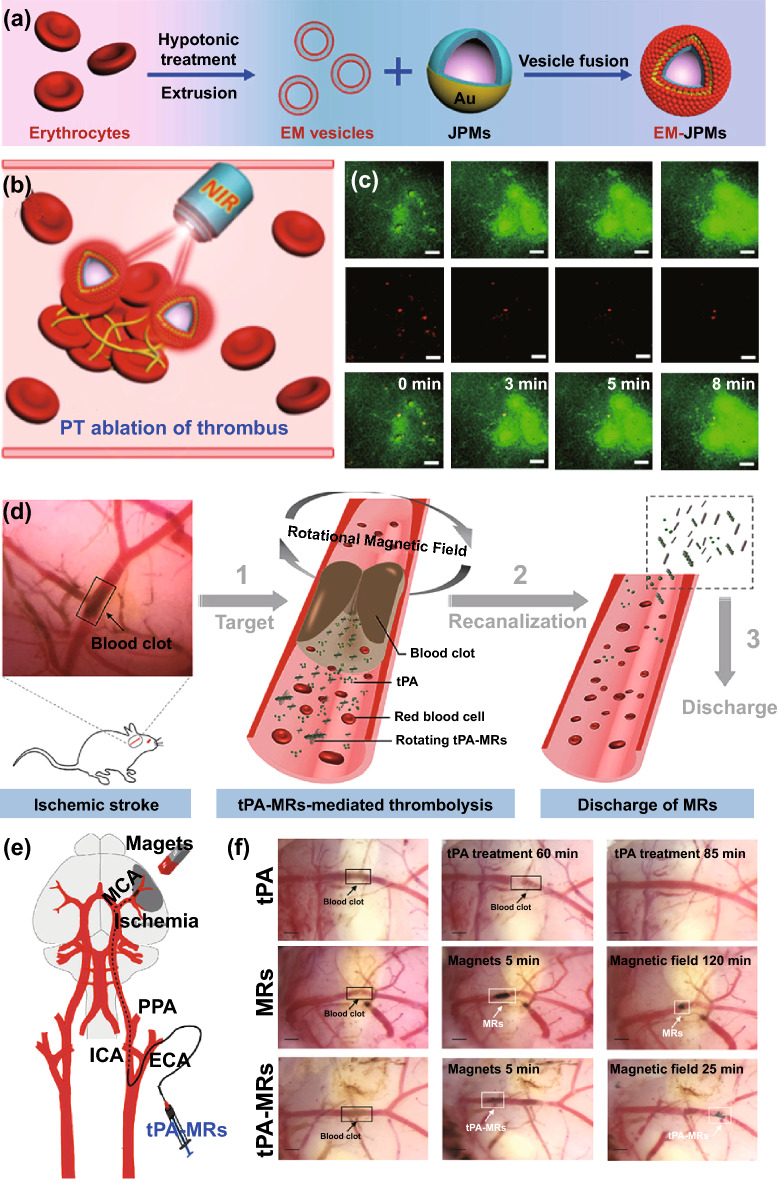
Schematic illustration of **a** EM-JPMs, and **b** the PT ablation of thrombus. **c** Time lapsed TP-CLSM fluorescence images of thrombus after treated with PT ablation procedure [119]. Copyright 2018 Creative commons. Schematic illustration of **d** the magnetic field responsive thrombolysis strategy, and **e** magnetic guidance tPA-MRs administration of through ICA to MCA. **f** The optimal cutting temperature compound (OCT) images of thrombolysis after treated with tPA, MRs under a rotational magnetic field and tPA-MRs under a rotational magnetic field [126]. Copyright 2018 American Chemical Society

Light has also been reported to drive some Janus micro-/nano-motors by generating kinetic energy, which could benefit tissue-selective drug delivery [119–123]. Blood clots are dense tissues with various cells, fibrin, and other ingredients, so it is of great significance for thrombolysis to penetrate into the interior of blood clots. In 2018, Shao et al. [119] prepared a kind of Janus polymeric motor (JPM), in which the Au layer was partially coated onto the surface of the capsule. JPMs were further modified with erythrocyte membranes (EM-JPMs) for biomedical applications (Fig. 9a). Under external NIR laser irradiation, the EM-JPMs showed obvious light-induced movement, and the trajectories were extended, which could benefit the collision with thrombus (Fig. 9b). The EM-JPMs could be propelled by the self-thermophoresis effect during NIR laser irradiation.

Human fibrinogen has been used as a thrombus mode to study the thrombolysis efficiency of engineered micro-motors. When performing NIR laser irradiation, the micro-motors could collide more often with the thrombus, and the aroused photothermal ablation could treat thrombus (Fig. 9c). A magnetically powered micro-motor was also designed, which acted as a carrier of thrombolytics [124, 125]. With irradiation by external magnetic fields, the micro-motors could penetrate the blood clots and deliver thrombolytics. In 2018, Hu et al. [126] prepared porous Fe_3_O_4_-microrods (MRs) and loaded them with the tPA. As shown in Fig. 9d, the tPA-MRs could rotate under an external rotating magnetic field, inducing mechanical lysis. Combined with the function of loaded tPA, tPA–MRs could show a thrombolysis effect. When used to treat an ischemic stroke model, an external magnet was applied, and the tPA–MRs could target blood clots after their injection (Fig. 9e). The clot lysis efficiency was significantly enhanced after treated with an external rotating magnetic field (Fig. 9f). However, the large size of the irradiation-derived micro-motor limited the applications in vivo, and further preparation of the nano-motors is necessary for this strategy.

## Conclusions and Perspectives

Nowadays, more than 200 nano-medicine-based products have been approved for clinical treatment or are undergoing clinical investigations. Among nano-medicine methods, liposomes are the most widely studied due to their good biocompatibility and biodegradability, and many liposome-based nano-medicines have been approved by FDA (e.g., Doxil, Ambisome, DepotDur, and DaunoXome). The pursuit of delivery strategies with higher efficiency and potential clinical applications is still on the way. Combined with in vivo imaging systems, drug delivery systems, and irradiation responsive systems, the advanced thrombosis systems should contain three factors:

Firstly, the accumulation of nano-medicines at thrombotic sites is a prerequisite for clinical treatment. Unlike the case of nano-medicines widely applied for tumors or inflammation, thrombotic sites are distributed in the blood flow, and the blood clots are very small. For this propose, it is of great importance to modify nano-medicines with the targeting antibodies or peptides, such as single-chain antibody (scFv), cyclic Arg-Gly-Asp (cRGD) peptide. However, phagocytosis of nano-medicines by macrophages could limit their blood circulation time. Thus, reasonable modifications to extend half-time are useful for drug accumulation, such as cell membrane biomimetic methods. Meanwhile, the morphology, size, and surface hydrophilic modulation also influence the biodistribution of nano-medicines.

Secondly, nano-medicines could gather in many tissues/organs in the body, and unwanted tissue damages must be considered. Thrombolytics could induce hemorrhage in the undesirable tissues or sites and even induce other cardiovascular diseases or organ damages. The delivery systems that responding to the thrombus microenvironment or photo/sound/magnetic irradiation could only release their cargoes in thrombotic sites, and only a minor amount of drug was leaked in normal tissues. A responsive design should strongly associate with the thrombosis microenvironment, and the microenvironment might simultaneously be changed, resulting in a combined therapeutic effect. The direct cell apoptosis induced by photo/sound/magnetic therapy could also benefit for the thrombosis treatment.

Thirdly, real-time imaging not only differentiates blood clots from other normal tissues, but also provides detailed information about thrombus. Thus, imaging-guided treatment is a key factor for personalized treatment.

With the development of nano-medicines, many thrombosis treatments have been reported, and some of them have obtained good thrombolysis effects with reduced biotoxicity. Up to now, most of clinical thrombolysis trials have been carried out on sonothrombolysis, showing obviously improvements in drug efficacy. However, there are some reasons responsible for the limitations of nano-medicines in clinical translation: (1) the advanced thrombosis systems often involve complex synthesis and purifications. (2) The therapeutic effect of nano-medicines may be inconsistent between animal models of thrombosis and human patients; (3) nano-medicines could gather in various organs, leading to unwanted side effects. The elimination of nano-medicines for thrombosis treatment in other healthy organs is encouraged to achieve the future success, and it would be even better to develop a nanosystem that can be cleared through kidney after the treatment. Considering the promising potential of nano-medicines for thrombosis treatment, much more efforts should be made to shorten the extensive procedures involved basical and clinical research. Successful translation of nano-medicines to the clinical thrombosis treatment will enable novel medical diagnostics and therapy to manage thrombosis for personalized medicine.
